# Understanding Adherence to Digital Health Technologies: Systematic Review of Predictive Factors

**DOI:** 10.2196/77362

**Published:** 2025-11-17

**Authors:** Teodora Figueiredo, Leovaldo Alcântara, Joana Carrilho, Constança Paúl, Elísio Costa

**Affiliations:** 1 RISE-Health, Competence Center for Active and Healthy Ageing, Faculty of Pharmacy, University of Porto, Rua Jorge de Viterbo Ferreira 228, Porto, 4050-313, Portugal, 351 914573768; 2 School of Medicine and Biomedical Sciences, University of Porto, Porto, Portugal; 3 RISE-Health, Department of Behavioural Sciences, School of Medicine and Biomedical Sciences, University of Porto, Porto, Portugal

**Keywords:** digital health technologies, adherence, adoption, engagement, acceptability, theory, model, framework

## Abstract

**Background:**

Digital health technologies (DHTs) are transformative solutions for health care challenges; however, sustaining long-term adherence remains a significant barrier, limiting their effectiveness.

**Objective:**

This systematic review aims to identify and categorize factors influencing adherence to DHTs and to identify theoretical foundations used to predict it.

**Methods:**

This review was conducted according to the PICO (population, intervention, comparison, outcome) strategy and followed the Cochrane Handbook and PRISMA (Preferred Reporting Items for Systematic Reviews and Meta-Analyses) 2020 guidelines. The protocol was prospectively registered on PROSPERO (CRD42024628168). Literature searches were performed in December 2024 in PubMed, PsycINFO, Scopus, and IEEE Xplore for studies published between 2019 and 2024 in English, Portuguese, or Spanish. Studies were eligible if they investigated factors influencing adherence to DHTs or theoretical foundations and tools predicting adherence. Nonpeer-reviewed studies, study protocols, and studies that did not explicitly report adherence outcomes were excluded. Risk of bias was assessed using the Joanna Briggs Institute Critical Appraisal Tools. Data were synthesized narratively through inductive thematic analysis, with factors influencing adherence extracted and categorized.

**Results:**

In total, 61 studies were included, mostly quantitative and conducted in Europe and North America. The populations were mainly patients with medical conditions, and most studies focused on mobile health apps. Study quality was moderate to high. The findings highlight a complex and multifaceted range of factors influencing adherence, which were categorized into four key domains: (1) personal factors (sociodemographic characteristics, health status, user characteristics, and personal beliefs and perceptions), (2) technology and intervention content factors (infrastructure and accessibility, user experience and performance, and content and features of the intervention), (3) social and support system factors (family and informal support and health care professional support), and (4) contextual factors. Among the theoretical foundations identified, the Unified Theory of Acceptance and Use of Technology (UTAUT) emerged as the most frequently applied.

**Conclusions:**

The findings highlight the need for integrative, health-specific models that combine behavioral, technological, and clinical aspects. Future research should focus on developing standardized adherence metrics and exploring the interactions between these factors to improve predictive models. However, the evidence base is limited by heterogeneity in study designs and adherence definitions, potential publication, and language bias.

## Introduction

Digital health technologies (DHTs) are increasingly being integrated into formal care pathways to address critical gaps in health care delivery. Digital health can be defined as the strategic use of technology to improve individual and population health, as well as patient care, through the integration of clinical and genetic data. It leverages advanced tools, such as telemedicine, wearable devices, virtual reality, and artificial intelligence to revolutionize the delivery of health care. By enabling real-time monitoring, personalized interventions, and seamless communication, DHTs promote a holistic, patient-centered, and interoperable model of care [1,2]. Since 2020, for example, German physicians and psychotherapists have been able to prescribe approved digital health apps as part of standard treatment [3].

However, sustaining the use of DHTs within routine practice goes beyond clinical validation and regulatory approval [4]. To ensure long-term effectiveness, the scientific community must now turn its attention to a longstanding challenge in health care, that is, adherence. Despite the demonstrated benefits, growing scientific evidence, and increasing availability of DHTs [5], adherence rates remain variable and, in some cases, unexpectedly low [6-10]. This is particularly evident with mobile health (mHealth) apps, which often experience high dropout rates over time, with a significant proportion of users failing to adhere to them as intended [8].

Adherence is a critical determinant of the success or failure of DHT implementation. Defining adherence in the context of DHTs is more complex than in traditional health care interventions, such as medication adherence, where there is often a clear “optimal dosage” [11]. Due to this complexity, some definitions in the literature focused narrowly on the extent to which individuals engage with the content of the DHT [12]. Alternatively, several authors have proposed the concept of intended use, that is, using the technology that was designed to be used, acknowledging that different DHTs may require different usage patterns to be effective in producing health outcomes [6,13-15]. This perspective aligns with the conceptualization of the World Health Organization (WHO), describing adherence as the degree to which individuals consistently adopt and integrate new technologies into daily processes, using them according to their functionalities and expected benefits. Based on this definition, adherence can be understood across 5 key dimensions, similar to adherence to domains, such as diet, medication, or physical activity. These domains are initial adoption (starting use), consistency and duration (sustained engagement aligned with intended use), dropout (premature discontinuation), and intensity (depth or frequency of use) [13,16,17]. Accordingly, this conceptualization will guide how adherence is defined, analyzed, and interpreted in this review.

Two related but distinct concepts are engagement and acceptance. Engagement refers to how individuals use and interact with the DHT, irrespective of the intended use. Engagement data can be used to measure or predict adherence to DHT [18,19]. Acceptance, on the other hand, pertains to users’ attitudes, intentions, and perceptions toward DHT, reflecting their willingness to use these technologies, an important predictor of adherence [20,21]. While acceptance predicts initial adoption, it may not sustain long-term adherence without ongoing engagement [22]. Engagement behaviors, on the other hand, directly reflect adherence consistency, duration, dropout, and intensity [23]. For these reasons, in this review, both engagement and acceptance are treated as predictors of adherence behavior.

Research on adherence to DHTs in real-world settings is limited, particularly concerning evidence-based barriers and facilitators, as well as individual, intervention, contextual, and clinical factors influencing adherence [8]. Understanding the factors that influence adherence to DHTs and developing robust tools to predict it is essential to maximize their effectiveness and long-term impact. Despite its importance, no specific model or standardized scale currently exists that can reliably predict adherence to DHTs. To date, potential adherence has typically been assessed using pre-existing theories, models, and frameworks retrieved from other areas, for instance, the technology acceptance model (TAM) and the unified theory of acceptance and use of technology (UTAUT) [24]. Behavioral theories, such as the theory of planned behavior (TPB) and the health belief model (HBM) have also been used to guide the design and evaluation of DHTs [24]. However, there is growing concern about the applicability and sufficiency of these approaches. These models are often applied in a formulaic manner and have limitations. Specifically, they tend to overlook the full spectrum of factors that influence adherence within health care contexts and fail to account for the entire variance in users’ behavioral intentions. Many existing frameworks also neglect important elements, such as health-related motivations, clinical and organizational contexts, and the degree to which technology is meaningfully integrated into users’ daily routines for long-term condition management [4,25]. More recently, frameworks originally developed for adherence to pharmacological treatments, such as the ABC taxonomy of adherence stages, developed by the European ABC Project (Ascertaining Barriers to Compliance), have begun to be explored for their potential adaptation to DHT adherence [26].

On the other hand, within the industry, particularly, most DHTs are designed without integrating theory-based strategies, models, or frameworks, or by applying them incorrectly [27]. The development process for DHT is often fragmented, lacking coordination and consensus on best practices for design. Furthermore, no single framework has been proven superior for designing effective DHT, leaving a gap in standardized guidance for creating DHT [28].

In response to the challenges identified, this systematic review aims to identify and categorize the factors that influence adherence to DHT. It also identifies existing theoretical foundations and tools used to predict adherence to DHT. By integrating and systematizing these key concepts, the review seeks to offer a holistic understanding of adherence to DHTs and how it can be effectively enhanced, providing valuable insights to inform the future development of more accurate adherence models.

To our knowledge, this is the first systematic review to evaluate all factors influencing adherence across diverse population groups and all types of DHT solutions. While previous systematic reviews have focused on specific contexts, none have provided a unified analysis encompassing the full spectrum of DHT solutions and user populations. They were limited by population, DHT type, or the scope of factors analyzed. For instance, 1 review [8] examined intended use, actual use, and factors influencing adherence, including 99 studies. It identified intervention- and patient-related factors that positively influenced adherence to mHealth apps for the prevention and management of noncommunicable diseases. A systematic review and meta-analysis assessed the design and implementation characteristics of eHealth tools adhered to by vulnerable groups (including older adults, chronically sick people, minorities, people with low socioeconomic status, and migrants), including 29 studies [6]. Another review synthesized findings on users’ perceptions of barriers and enablers to adhering to digital health interventions among older patients with cancer, including 5 studies [29]. Finally, a review focusing on the prepandemic period of SARS-CoV-2 examined predictors of adherence to mindfulness-based eHealth interventions, including 69 studies, identifying demographic or personal and psychological predictors [30].

## Methods

### Overview

The protocol for this systematic review was registered a priori on PROSPERO under the registration ID number CRD42024628168 in December 2024. It adhered to the guidelines outlined in the Cochrane Handbook for Systematic Reviews of Interventions [31], and it also followed the PRISMA (Preferred Reporting Items for Systematic Reviews and Meta-Analyses) checklist [32]. The PICO (population, intervention, comparison, outcome) strategy was used to formulate the research question and search strategy [33].

### Research Questions

This study aimed to address the following research questions (RQs):

1. RQ1: What factors influence user adherence to DHTs?
2. RQ2: What are the existing theoretical foundations and tools (standardized scales or predictive instruments) that have been used to predict adherence to DHTs?

### Review Objective

The primary objective of this review is to identify, synthesize, and categorize the factors that influence adherence to DHTs, including both barriers and facilitators across diverse populations and settings. In addition, the review aims to examine the theoretical foundations and tools that have been applied to predict adherence. By integrating these elements, the review seeks to provide a comprehensive understanding of how adherence to DHTs can be improved and inform the development of more targeted interventions and more accurate adherence models, highlighting existing gaps in the current evidence.

### Search Strategy and Screening Process

In December 2024, data were extracted from 4 different electronic databases, namely PubMed, PsycINFO, Scopus, and IEEE Xplore. The search strategies combined controlled vocabulary (MeSH [Medical Subject Headings] terms in PubMed) and free-text keywords related to (1) models, frameworks, and determinants (eg, tool, measure, questionnaire, survey, instrument, scale, theoretical model, framework, conceptual model, determinant, predictor, barrier, and facilitator); (2) adherence and related terms (eg, adherence, compliance, persistence, nonadherence, and dropout); and (3) DHTs (eg, digital health, eHealth, mHealth, telemedicine, and health apps). Boolean operators (AND, OR) and truncation were used. The full search strings for each database, including applied syntax, are provided in Multimedia Appendix 1. Filters were applied in all databases to include only studies published in the last 5 years and written in English, Portuguese, or Spanish. Additionally, the search was restricted to studies involving humans. The search was not updated after December 2024.

After duplicate removal, all extracted studies were independently assessed by 2 reviewers, TF and LA, following a 3-step process: title screening, abstract screening, and full-text review. The screening process was conducted using the Rayyan platform [34], which allows blinded, independent screening. Disagreements between reviewers were resolved through discussion and, when necessary, with input from a third reviewer (EC). The agreement between the 2 independent reviewers was assessed using Cohen Kappa (κ) coefficient. Decisions to include or exclude studies were coded as binary (include=1, exclude=−1). The Kappa value was calculated for the 3 screening phases. In the title phase, the resulting value was 0.22, indicating only fair agreement according to Landis and Koch’s [35] classification. In the abstract screening phase, the κ value was 0.62, indicating substantial agreement between reviewers, and the full-text phase was 0.42, indicating moderate agreement.

When assessing the titles, studies that met one or more of the exclusion criteria listed below were not considered:

1. Studies not addressing DHTs, models, theories, frameworks, tools, or factors influencing DHT adherence or adherence-related concepts (such as adoption, consistency, duration, dropout, intensity, acceptability, or engagement)
2. Studies not published in English, Portuguese, or Spanish

During the abstract and full-text analysis, the selection was based on the criteria presented in Textbox 1.

Textbox 1. **Inclusion and exclusion criteria for the screening of abstracts and full texts.**

**Inclusion criteria**

- Study design: experimental studies, observational studies, qualitative studies, mixed methods studies, intervention studies, implementation science studies, case studies, and systematic reviews/meta-analyses or scoping reviews
- Population: all participants (eg,individuals using digital health technology [DHT] for prevention, diagnosis, monitoring, or treatment or health care professionals)
- Exposure: studies investigating adherence to DHT (ie, studies that used adherence as an outcome measure or studies that explicitly stated how other outcome measures are used as a proxy for adherence—adoption, consistency, duration, dropout, intensity, acceptability, or engagement)
- Outcome: studies reporting factors influencing adherence or related concepts (adoption, consistency, duration, dropout, intensity, acceptability, or engagement) and tools, theoretical models, or frameworks related to predicting or explaining adherence to DHT
- Publication: studies published in peer-reviewed journals

**Exclusion criteria**

- Study protocols, theoretical papers, conceptual papers, and position papers/studies not peer-reviewed (eg, thesis and conference abstracts)
- Studies not addressing adherence or adherence-related concepts (adoption, consistency, duration, dropout, intensity, acceptability, or engagement) to DHT
- Studies focused only on the use, usability, or technical development of DHT without explicitly connecting these aspects to adherence
- Studies that do not mention factors that influence, explain, or predict adherence or adherence-related concepts (adoption, consistency, duration, dropout, intensity, acceptability, or engagement)
- Studies in which adherence and related constructs (eg, adoption, use, and engagement) were used ambiguously or interchangeably without a clear definition or distinction, making it impossible to interpret the reported outcomes as measures of adherence or adherence-related behavior
- Studies for which the full text was not available

### Quality Assessment

The methodological quality of the included studies was independently evaluated by 2 reviewers (TF and LA) using the Joanna Briggs Institute (JBI) Critical Appraisal Tools, with specific validated checklists applied according to each study design [36]. The JBI Critical Appraisal Tools were selected for this review, as they are designed to be used across a variety of study types within systematic reviews, enabling consistent quality appraisal, and are widely adopted in health research [37].

Each study was assessed using the appropriate JBI checklist, with items scored as 1 (“Yes”) or 0 (“No,” “Unclear,” or “Not applicable”). The overall quality score was calculated as the proportion of “Yes” responses relative to the total number of items. Based on these scores, studies were classified as high quality (≥75%), moderate quality (50%‐74%), or low quality (<50%). A summary of the final quality assessment scores and categorizations is provided in Multimedia Appendix 2.

### Data Extraction and Content Analysis

Data from the selected studies were extracted by TF and LA using a structured Microsoft Excel spreadsheet, capturing key details such as authors, year of publication, country, study design, adherence or adherence-related concept, validated theories, models, or frameworks, population characteristics, type and purpose of the DHT, factors influencing adherence, and how adherence or adherence-related concept was defined or measured.

Given the inclusion of different study designs, the data extraction process varied accordingly:

1. Quantitative studies: Only factors influencing adherence or related concepts that showed a statistically significant association were extracted.
2. Qualitative studies and reviews: Facilitators and barriers to adherence were extracted based on their positive or negative influence on adherence. Additionally, factors influencing adherence that do not explicitly fit the classification of barriers or facilitators were also extracted when applicable. These include factors that may impact adherence positively or negatively.

These data are summarized in Multimedia Appendix 3.

Factors influencing adherence were initially identified through an inductive thematic analysis [38], allowing themes to emerge naturally from the data without imposing a predefined framework. Both reviewers (TF and LA) were involved in all stages of the analysis. They independently coded the data, identified patterns, and grouped similar factors into descriptive categories and subcategories. Any discrepancies were discussed and resolved through consensus, ensuring the reliability and validity of the categorization process. While the initial coding was data-driven, the development and refinement of the final categories were also informed by theoretical models and frameworks commonly used in the field, such as TAMs and health behavior theories. This combined approach allowed the categories to remain grounded in the data while also aligning with established theoretical constructs, thereby enhancing the clarity and interpretability of the findings.

A meta-analysis was not performed due to the substantial heterogeneity of the included studies. The studies varied significantly in their design (qualitative, quantitative, mixed methods, and reviews), populations (including patients with different conditions, health care professionals, and the general public), types of DHT (including apps, telehealth, and wearable devices), and the adherence-related concepts investigated. Furthermore, measurement approaches differed widely (including using validated scales, usage metrics, or qualitative findings). This conceptual and methodological variability limited the feasibility of statistical synthesis. A systematic mapping of these differences is presented in Multimedia Appendix 3. Therefore, a structured narrative synthesis was conducted to summarize and categorize the findings.

## Results

### Overview

**Figure 1.**
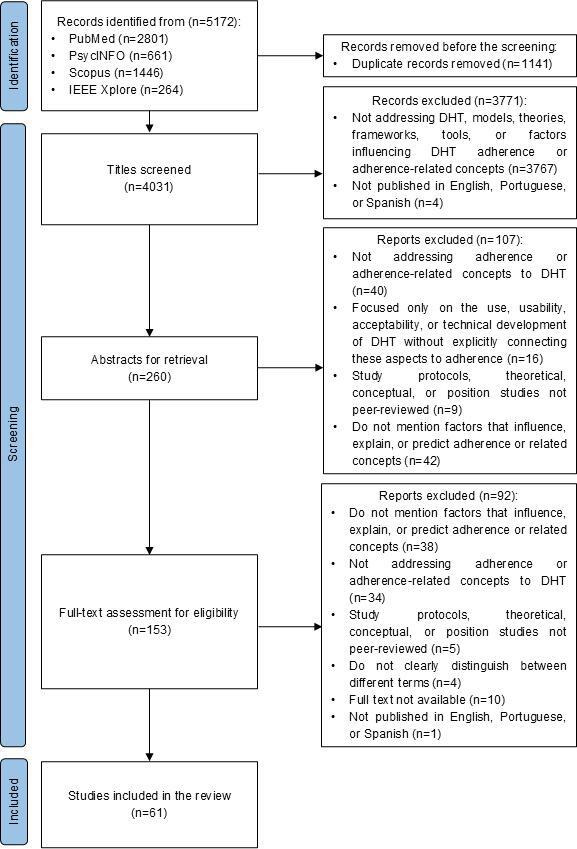
Studies’ screening process based on the PRISMA (Preferred Reporting Items for Systematic Reviews and Meta-Analyses) flowchart. DHT: digital health technology.

A total of 4031 studies were identified through searches in 4 databases (PubMed, PsycINFO, Scopus, and IEEE Xplore) after removing duplicates. Following the screening process, which applied predefined inclusion and exclusion criteria, 61 studies [6,8,25,29,30,39-94] were deemed eligible and included in this systematic review. The detailed selection process is illustrated in the PRISMA flow diagram (Figure 1).

### Characteristics of the Included Studies

Table 1 summarizes the characteristics of the included studies, while Multimedia Appendix 3 presents each study individually in detail. The majority of studies were quantitative (n=32; [39,42,44-46,48,49,53,54,58-67,71-75,78,80-82,87,88,90,93]), followed by qualitative studies (n=15; [41,47,51,52,57,68,70,76,77,79,83,85,86,89,94]) and 4 studies [43,50,55,69] used mixed methods approaches. Among the qualitative studies, the most commonly used data collection method was semistructured interviews (n=7; [41,47,51,70,76,77,83]), followed by focus groups (n=2; [52,79]), in-depth interviews (n=2; [86,94]), questionnaires (n=2; [68,85]), pre-post interviews (n=1; [89]), and think-aloud interviews (n=1; [57]) to identify factors predicting adherence. Additionally, this review also included 6 systematic reviews [6,8,29,30,56,84] and 4 scoping reviews [25,40,91,92]. The studies included in this review cover various geographical regions. The majority were conducted in Europe (n=25; [43-45,49-51,53,57,58,62,65-67,69,71,74-77,79,81,83,85,88,90]), followed by North America (n=14;[39,42,48,52,54,59,61,63,70,72,73,78,80,89]), Asia (n=7; [46,47,55,60,86,93,94]), Oceania (n=4; [41,68,82,87]), and Africa (n=1; [64]). Additionally, several studies involve multiple countries worldwide (n=10; [6,8,25,29,30,40,56,84,91,92]).

**Table 1.** Descriptive characteristics of the included studies (N=61).

Characteristics		Studies, n
Type of study		
Quantitative		32
Qualitative		15
Mixed methods		4
Systematic review		6
Scoping review		4
Geographical region		
Europe		25
North America		14
Asia		7
Oceania		4
Africa		1
Multinational		10
Concepts studied		
Adherence		20
Engagement		11
Adoption		12
Acceptance		9
Compliance		4
Desirability, acceptability, and adherence		2
Intention to continue use		1
Continue use		1
Persistence		1
Discontinue the use		1
Dropout		1
Study population		
Patients/people with a medical condition		36
Adults (≥18 years)		14
Health care professionals		9
Students		2
Caregivers		1
Vulnerable groups		1
Pregnant women		1
Adolescents		1
Type of DHT^a^		
mHealth^b^ apps		31
Telehealth solutions		7
Text message–based interventions		6
Web-based programs		3
Conversational agents		1
Cell phone-based interventions		1
mHealth solutions		3
DHTs		1
eHealth		2
Digital health interventions		2
Wearables		2
Ingestible microsensor		1
Digital pillbox		1
Definition/measurement of adherence or adherence-related behavior		
No clear definition/measure		22
With definition/measure		39
Methodological quality		
High quality		25
Moderate quality		36
Low quality		0

a DHT: digital health technology.

b mHealth: mobile health.

The studied concepts included adherence (n=20; [6,8,29,30,40,48,51,53,55,57,58,64,68,74,75,79,81,91-93]) and adherence-related concepts, such as engagement (n=11; [39,41,42,44,52,61,63,69,78,82,87]), adoption (n=11;[25,46,47,50,59,60,65,73,76,77,83]), acceptance (n=9; [43,56,70,84-86,88,90,94]), compliance (n=4; [45,49,60,71]), desirability, acceptability, adherence (n=2; [66,67]), intention to continue use (n=1; [54]), continued use (n=1; [89] persistence (n=1; [80], discontinuation of the use (n=1; [72]), and dropout (n=1; [62]).

The majority of the included studies focused on patients or people with a specific medical condition (n=36; [29,39-44,46-49,51,53,55-57,60-67,70-72,75,79,81,83,84,86,93,94]), with patients with diabetes being the most frequently studied group (n=8; [43,44,46,47,61,63,66,67]). Other common populations included patients with cancer (n=6; [29,40,49,51,60,81]), individuals with cardiovascular conditions (n=5; [39,53,55,75,93]), and patients with chronic conditions (n=5; [48,62,71,79,83]). Several studies included adults in general (n=14; [8,52,54,59,68,69,74,80,82,85,88-90,92]) and health care professionals (n=9; [25,50,56,58,73,76,77,86,94]), such as general practitioners, physicians, psychotherapists, psychiatric prescribers, and other health care providers. One study [65] also included caregivers.

A variety of DHTs were examined in the included studies. The majority focused on mHealth apps (n=31; [8,40,44-53,57-60,65,68-71,74,76,77,79,81,87-91]), followed by telehealth solutions (n=7; [54,56,66,67,75,78,80]), text message–based interventions (n=6; [42,43,55,63,64,82]), and web-based programs (n=3; [41,72,84]). Additionally, some studies investigated conversational agents (n=1; [39]) and cell phone–based interventions (n=1; [86]). A few studies explored broader categories of digital health, including general mHealth solutions (n=3; [25,61,92]), DHTs (n=1; [83]), eHealth (n=2; [6,30]), and digital health interventions (n=2; [29,62]). Fewer studies focused on digital devices, such as wearables (n=2; [85,93]), an ingestible microsensor (n=1; [73]), and a digital pillbox (n=1; [94]). The identified DHTs serve various health care purposes that can be categorized into chronic disease management, health promotion, education and disease prevention, mental health and well-being, telemedicine and remote monitoring, and condition-specific digital interventions.

A part of the studies did not report a clear definition or measure of adherence or the adherence-related concept (n=22; [25,29,41,46,47,50-52,54-57,59,68,70,75-77,79,83,86,94]). Among those that did, definitions and measurements varied widely. Some studies assessed adherence through frequency and duration of app or system use, while others focused on interaction with digital content, response rates to questionnaires, completion thresholds, or patterns of sustained engagement. Several studies also relied on self-reported data.

The quality appraisal revealed that 25 of the included studies [39,45-47,53,54,56-60,64-67,73,75,76,78,80,81,87,88,90,93] were classified as high quality and 36 [6,8,25,29,30,40-44,48-52,55,61-63,68-72,74,77,79,82-86,89,91,92,94] as moderate quality (Multimedia Appendix 2). No studies were rated as low quality, indicating an overall satisfactory methodological standard across the body of evidence.

### Factors That Influence Adherence (or Adherence-Related Concepts)

#### Overview

**Figure 2.**
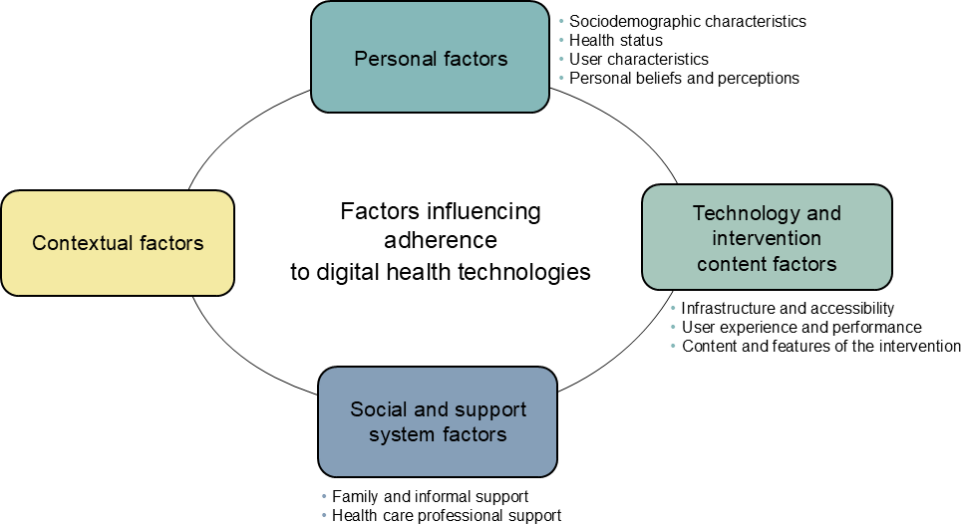
Categories and subcategories of influencing factors of adherence to digital health technology.

Given the wide range of extracted factors and their varying relationships with adherence found in the included articles, factors were grouped into 4 categories (Figure 2), including personal factors, technology and intervention content factors, social and support system factors, and contextual factors. These categories were informed by theoretical models and frameworks commonly applied in the field. Specifically, personal factors reflect constructs from health behavior theories, such as the HBM [95] and protection motivation theory (PMT) [96]. Technology and intervention content factors were informed by technology acceptance frameworks: TAM [97] and UTAUT [22]. Social and support system factors draw on concepts from social cognitive theory (SCT) [98] and UTAUT [97]. Contextual factors were considered in light of broader implementation frameworks, such as the diffusion of innovations (DOIs) theory [99].

Among the categories, personal factors were the most frequently reported (44 studies; [8,29,30,39-42,44-49,52,53,55,56,58,59,61-64,66-69,71-73,75-81,83,87-92]), followed by technology and intervention content factors (26 studies; [6,8,25,29,41,43,46,47,51,52,54,57,60,68-70,77,82-86,89,91,92,94]), contextual factors (14 studies; [50,57,59,64,65,68,69,74,86,88,90-92,94]), and social and support system factors (11 studies; [8,50-52,56,57,65,75,83,86,94]). Table 2 presents a cross-tabulation of these key factors by DHT type, providing a quantitative overview of the most consistently reported predictors across studies. Each category’s key factors are briefly summarized in the next sections. It is important to take into account that some variables showed mixed results in predicting adherence.

**Table 2.** Number of studies reporting each factor category by type of digital health technology.

Category	mHealth^a^ apps	Telehealth solutions	Text message–based intervention	Other DHTs^b^	Total
Personal factors, n	25	7	5	7	44
Technology and intervention content factors, n	13	0	2	11	26
Contextual factors, n	8	0	1	5	14
Social and support system factors, n	5	2	0	4	11

a mHealth: mobile health.

b DHT: digital health technology.

#### Personal Factors

Factors included in this category are sociodemographic characteristics, health status, user characteristics, and personal beliefs and perceptions. Several included reviews and qualitative studies put personal factors as important predictors, barriers, or facilitators of adherence, including sociodemographic variables (such as age, gender, education level, relationship status, and employment status), user-related characteristics (such as personal circumstances and digital literacy), and health status (cancer-related factors, presence of comorbidities, mental health, health condition–related factors, and psychological predictors) [8,30,40,41,55,68,91,92].

Regarding sociodemographic characteristics, age was shown to be a relevant predictor in several studies. In these studies, increasing age was shown to positively influence adherence, negatively influence it [39,44,48,53,61,69,80,88], or have mixed effects [62]. Similarly, being a woman was shown to either positively [52,59,73,87] or negatively [44,75] influence adherence. Level of education was also a significant predictor, generally promoting adherence [45,61,62,72,75], except in 1 study [64]. Regarding socioeconomic status, lower financial deprivation [88] and higher monthly or annual household income had a positive effect on adherence [45,61,90]. Ethnicity also played a role, with its impact on adherence varying by population and country [39,45,61,63,78,90]. Finally, 1 study [30] found that single or divorced patients had lower adherence compared to married or partnered individuals.

Regarding health status, disease history, and management influenced adherence [66]. Better disease control was identified as a predictor of higher adherence [43], while a greater comorbidity burden positively affected adherence to telehealth among patients at risk of hospitalization [78]. Similarly, previous atrial fibrillation ablation was associated with greater adherence to an mHealth app for managing atrial fibrillation care [53]. Mental health conditions also play a role in adherence. Severe depression and anxiety were linked to reduced adherence to telemedicine [66], while high perceived stress was identified as a barrier to mHealth adherence [69]. Diagnosis of depression, obesity, and dyspnea significantly negatively influenced adherence [71], and being overweight or obese was also associated with lower adherence [69]. Additionally, lower severity of anxiety symptoms was found to negatively impact adherence to an electronic mental health treatment program [72]. In the same manner, decreased distress negatively affected adherence to a mindfulness app, and vice versa [81]. Physical impairments can further affect adherence. Not having a disability, handicap, or chronic disease was linked to better adherence to telemedicine [80]. Conversely, vision problems were found to reduce adherence to an mHealth app in both a quantitative [46] and qualitative study [47].

Concerning user characteristics, smoking was significantly associated with lower adherence [45,69,71,90]. Poor or lack of digital literacy also had a negative impact on adherence [46], and low self-perceived digital competence and digital literacy were commonly identified as barriers [47,76,83,86]. Other factors that negatively influenced adherence included low awareness, low motivation to learn, and time constraints [25,46,47,68]. Forgetfulness was specifically noted as a barrier among older adults using an app for cognitive decline prevention and detection [52]. Additionally, users’ persistence in pursuing health goals was identified as an influencing factor [89].

Personal beliefs and perceptions also affected adherence. Discomfort with video communication and difficulty interpreting nonverbal cues [80], personal attitudes toward technology [77], and illness perception [83] were found to negatively impact adherence. On the other hand, positive perceptions played a key role in promoting adherence. The perceived usefulness of DHT was highlighted as a significant predictor in multiple studies [29,54,56,60,68,83,88]. Similarly, perceived effectiveness [43] and perceived benefits [79] were also associated with better adherence.

#### Technology and Intervention Content Factors

This category includes factors related to technology infrastructure and accessibility, user experience and performance, and the content and features of the intervention. Included reviews highlighted several technological features as important predictors of adherence to DHTs [25,84,91,92], emphasizing the significant role that technology-related factors play in user adherence.

Regarding technology infrastructure and accessibility, the cost of DHTs, such as app subscriptions and device expenses, was frequently discussed as a barrier, negatively influencing adherence [25,43,46,47,77,84]. For mHealth solutions, limited access to phones and internet connectivity also hindered adherence, especially in middle-income countries [25,47,70,94], along with restricted smartphone storage [41,42]. Specific device-related limitations were also identified. For wearables, factors such as limited battery life, bulkiness, and device size were cited as barriers [85]. In the case of digital pillboxes, issues related to durability, portability, and storage were noted [94]. Additionally, ethical and privacy concerns associated with the use of DHTs were raised in several studies [77].

Regarding the user experience and performance subcategory, user experience was consistently highlighted as a key factor influencing adherence to DHTs. Several studies discussed how users’ perceptions of the intervention, its capabilities, benefits, and overall experience impacted their adherence [57,76,89]. Factors, such as prior exposure to similar content, feeling emotionally confronted by the material [41], or perceiving the intervention as useful [25,60] played a significant role in shaping user adherence. Ease of use [52,60,86], general usability [29], and user-friendliness, including whether the app functioned smoothly, were identified as key facilitators [8,70]. In contrast, technical issues, such as malfunctions, poor operation, and lack of stability, were reported as barriers that negatively affected adherence [68,69,77].

Finally, regarding the content and features of the intervention, several elements were identified as positively influencing adherence to DHTs. These included the personalization of content [8,84], coherent, and relevant information [43], and an appropriate choice of design features [51]. Multimodal interventions, offering engaging and interactive content [68], including interactive messages [82], and providing new knowledge [70,83], were seen as more effective in promoting adherence. Gamification elements, social interaction features, and the possibility for direct patient-provider communication also contributed positively [6]. Furthermore, easy and enjoyable practical challenges [68], opportunities for feedback [57], and audible or visual reminders [86] were highlighted as important facilitators. Interventions were more successful when they were time-efficient and required minimal or no skill acquisition to engage with [84]. On the other hand, some features were identified as barriers to adherence. These included certain notification or reminder styles, particularly when used with high frequency [82,85], and the excessive length of video content [68].

#### Social and Support System Factors

This category was divided into 2 subcategories, namely family and informal support and health care professional support. Social influences and broader social context were consistently highlighted as important factors affecting adherence to DHTs [52,56,83].

Within the domain of family and informal support, having a supportive and involved family or strong social relationships was identified as a facilitator of adherence [51,86]. Additionally, community-based support was shown to contribute positively [75]. However, not all forms of social involvement were beneficial. One study described “parental meddling” as a barrier to adherence among adolescents using a DHT.

The role of caregivers was also emphasized. When a caregiver was involved in activating the app, adherence significantly increased [65]. Similarly, the presence of a caregiver with digital proficiency was positively associated with higher adherence levels [75].

The involvement of health care professionals and the patient-provider relationship was also found to influence adherence. Having a trusted prescriber was identified as a positive factor [51], and active engagement of health care professionals in patient care was seen as a facilitator [86]. In contrast, poor counseling or lack of explanation about the DHT from health care professionals was viewed as a barrier [86,94].

Health care professionals themselves were also influenced by their environment. Reports of positive experiences with DHTs from medical colleagues increased their willingness to adhere to these technologies [50]. On the other hand, inadequate training in the use of DHTs was a barrier, while better coordination among health care providers in using these tools was seen as a facilitator of adherence [86]. Moreover, many professionals perceived that DHTs enhanced the patient-provider relationship, improving both the quality and efficiency of care, which further encouraged adherence [86].

#### Contextual Factors

This category includes broader social, structural, and environmental influences that affect adherence to DHTs, factors that go beyond individual characteristics or the technology itself. Contextual factors were identified as relevant to adherence in 2 included scoping reviews [91,92].

Health care professionals reported as facilitators of adherence (1) when apps were endorsed by medical associations, (2) when they had the opportunity to test the tools, and (3) when clear information was provided about their use. Additionally, increased reimbursement for medical services related to DHTs was seen as a facilitator [50].

In public health contexts, such as during the COVID-19 pandemic, contextual variables significantly influenced adherence. Factors, such as concern about the pandemic, knowledge of virus transmission, and trust in political representatives were associated with higher adherence to contact-tracing apps [88]. Similarly, trust in government and health authorities and better compliance with public health measures, like mask-wearing, were linked to greater use of SARS-CoV-2 mitigation apps [90].

Cultural and social identity also played a role. Men who perceived themselves as highly masculine showed lower adherence to mental health apps [59], whereas women who had disclosed their HIV status adhered more to DHTs targeting the prevention of mother-to-child transmission [64]. Stigma within families, particularly related to diagnosis, was identified as a significant barrier in some cases [86,94].

### Theories, Models, and Frameworks Applied to Predict Adherence-Related Concepts

Several theories, models, and frameworks were used across the included studies to explain or support findings related to DHT adherence. The most frequently cited was the UTAUT (n=5; [55,56,76,86,94]), followed by the technology readiness and acceptance model (TRAM; n=2; [46,47]), HBM (n=2; [56,76]), theoretical framework of acceptability (TFA; n=1; [43]), PMT (n=1; [56]), technology readiness (TR; n=1; [56]), SCT (n=1; [56]), TAM (n=1; [56]), TAM2 (n=1; [56]), theory of interpersonal behavior (TIB; n=1; [56]), theory of planned behavior (TPB; n=1; [56]), theory of reasoned action (TRA; n=1; [56]), DOI (n=1; [56]). A specific tool was also developed and validated for assessing patients’ desirability, acceptability, and adherence to telemedicine in diabetes, the QtelemeDiab [66,67].

## Discussion

### Principal Findings

Sustained adherence to DHTs remains a major challenge, limiting their long-term effectiveness and impact. Research in real-world settings is still limited, especially regarding the factors that influence it. Gaining a better understanding of these factors is crucial to ensuring that DHTs reach their full potential. To address this, this systematic review aimed to identify, analyze, and categorize the factors influencing adherence to DHTs, while also exploring the theoretical foundations and tools applied to predict it. A comprehensive search across 4 databases identified 61 peer-reviewed studies [6,8,25,29,30,39-94] meeting predefined inclusion criteria. Data were extracted and thematically analyzed.

Regarding the factors influencing adherence (RQ1), the findings highlight a complex and multifaceted range of factors, which were categorized into 4 key domains, such as personal factors (sociodemographic characteristics, health status, and user characteristics), technology and intervention content factors (infrastructure and accessibility, user experience and performance, and content and features of the intervention), social and support system factors (family and informal support and health care professional support), and contextual factors.

It is important to take into account that some results, in particular sociodemographic characteristics, were inconsistent across studies, with age, gender, level of education, and ethnicity yielding mixed outcomes on adherence. These inconsistencies may be due to variations in study populations, contexts, types of DHTs, or the way adherence was defined and measured. This also suggests that sociodemographic factors likely interact with other personal, social, contextual, or technological variables, rather than exerting a uniform influence across settings. It is also important to consider the strength of evidence across studies of varying methodological quality. Among the 61 included studies [6,8,25,29,30,39-94], 25 [39,45-47,53,54,56-60,64-67,73,75,76,78,80,81,87,88,90,93] were rated as high quality and 36 [6,8,25,29,30,40-44,48-52,55,61-63,68-72,74,77,79,82-86,89,91,92,94] as moderate quality. Several factors, such as sociodemographic characteristics, digital literacy, motivation, perceived usefulness, and caregiver support, were consistently supported by high-quality studies, indicating strong evidence for their association with outcomes. In contrast, factors related to the content and specific features of the intervention were mainly reported in isolated or moderate-quality studies, and therefore, these associations should be interpreted with caution.

The findings of this review, together with current literature, indicate strong interactions both within and across domains, reinforcing the need for a systems-level perspective on adherence. For instance, an individual’s digital literacy (a personal factor) can significantly shape how they interact with a DHT’s interface (a technological factor), which in turn influences adherence, and this is particularly relevant in older populations (personal factor) [100]. Similarly, social support from health care professionals can help mitigate low user confidence or reduce perceived complexity of the intervention [51], and intervention features, such as reminders or gamification, can compensate for low intrinsic motivation [101].

Additionally, several theoretical foundations and tools (RQ2) were identified across the studies reviewed to explain adherence or adherence-related concepts. In general, the results indicate that technology acceptance theories, such as the TAM, UTAUT, TRAM, and TAM2, can provide a strong theoretical foundation to examine adherence-related behaviors. The frequent use of UTAUT in the reviewed studies may be attributed to its broader scope compared to TAM, as it offers a more holistic framework for studying acceptance. UTAUT proposes 4 core determinants (performance expectancy, effort expectancy, social influence, and facilitating conditions) [22].

However, some included studies acknowledged that these theoretical foundations remain insufficient on their own. Studies emphasized the need to expand these models by incorporating additional constructs, contextual factors, or previously unexamined external variables to better account for the complexity of adherence in digital health contexts [46,47] or that the use of these models could have potentially missed some findings that did not fit into this predetermined framework [86,94]. In fact, none of these models were originally designed specifically for DHTs; rather, they emerged from organizational and commercial contexts aimed at explaining initial technology uptake, not sustained engagement or long-term adherence in health care settings [22,102]. As such, they often fail to capture the complexity of health care systems, including patient-provider relationships, individual health motivations, and the influence of clinical environments [103,104]. In the same way, the TR index only considers 4 dimensions (innovativeness, optimism, insecurity, and discomfort) that collectively explain technology propensity and usage [105]. Additionally, the DOI, also identified in the included studies, offers a broader perspective on how innovations spread across populations [99]. This highlights the need for more comprehensive and health-specific models to better predict adherence behaviors.

On the other hand, some health-specific models like HBM and PMT mentioned in the included studies incorporate motivational and behavioral components relevant to health contexts [95,96]. However, these tend to be limited in addressing technological usability, personalization, and other factors inherent to digital health interventions. In addition to these, other models and theories related to health behavior were also mentioned in the included studies, such as SCT, TPB, TFA, TRA, and TIB. These frameworks bring important behavioral and cognitive elements, such as self-efficacy, intention, habits, and social norms, and are often used to understand and improve treatment adherence [106].

Only 1 study [67] proposed a specific tool (the QtelemeDiab) for assessing desirability, acceptability, and adherence to telemedicine among patients with diabetes. Although promising, the tool is limited to a specific population and type of technology, and its generalizability remains uncertain.

Notably, despite the relevance of theoretical guidance in understanding adherence, only 10 [43,46,47,55,56,66,67,76,86,94] of the included studies used any theoretical model or tool to explain their findings on adherence-related behaviors, highlighting a significant gap in the current literature. Overall, these findings underscore a critical gap. Most existing models focus on initial acceptance or psychological constructs, failing to fully address the complex, dynamic nature of adherence in digital health. There is a clear need for more integrative, health-specific, and context-sensitive models that combine behavioral, technological, and clinical dimensions. One possible direction for future model development would be to integrate components from both TAMs (such as UTAUT) and health behavior theories (such as HBM), as used in 1 included study [77], ensuring that dimensions identified in this review are systematically incorporated. This systems-level approach would enable more accurate predictions of adherence behaviors across populations, DHT types, and real-world settings.

Additionally, although this review included studies addressing adherence and related concepts such as engagement, adoption, acceptance, continued use, persistence, and dropout, it became evident that these terms were not consistently defined across the studies. Definitions of adherence and adherence-related behaviors varied, as did the methods and thresholds used to measure them. Moreover, several studies did not report any explicit definition of adherence. Among those that did, most conceptualized adherence in terms of intended use, actual use of functionalities as intended, and sustained use over an extended period of time, which goes in line with the definition adopted and defended throughout this systematic review, as explained previously. The persistent existing lack of consensus highlights the need for all researchers to adopt a common and clear definition and standardized measurement approaches for adherence and related behaviors. Indeed, these terms are sometimes used interchangeably in the literature, with one sometimes being used to define another, a concern previously highlighted by other authors [13,107].

This systematic review offers important insights for developers, health care professionals, and policymakers aiming to improve adherence to DHTs. For developers, DHTs should be designed with a user-centered approach that considers the diverse and evolving needs and characteristics of users. This includes incorporating features that enhance usability, personalization, and accessibility while also addressing technical barriers. Previous literature has also highlighted the value of cocreation, particularly co-design, in the development of DHTs [108]. While user-centered design considers users’ needs throughout the process, co-design goes further by actively involving users and stakeholders (eg, family, caregivers, and health care professionals) as partners during all stages of design. This ensures that solutions are grounded in real-life experiences and expectations. Co-design activities should be adapted to the characteristics of the target population and may include techniques, such as personas, user scenarios, ethnography, observations, interviews, and usability testing. Practical strategies, including incorporating features, such as reminders, gamification, or tailored feedback, can support adherence, particularly for users with lower intrinsic motivation or limited digital literacy. Integrating insights from both technology acceptance and health behavior models can better inform the design, deployment, and evaluation of interventions.

For health care professionals, patient adherence can be promoted by actively endorsing DHTs, explaining their benefits, addressing patient concerns, and providing hands-on guidance during initial use. Supportive interactions should consider social and contextual factors. Family or caregiver involvement can improve adherence, but care must be taken to avoid pressure or conflict. For policymakers and health care systems, efforts should focus on enabling environments that support both patients and providers. For instance, providing more robust training and support for health care professionals to enhance their confidence and competence in using DHTs, as well as using incentives for adherence to validated DHTs. This is an already well-discussed topic [109]. Policies should also consider strategies to integrate social support mechanisms safely and effectively into clinical practice. Taken together, these recommendations highlight the importance of a systems-level approach that integrates technological, personal, social, and contextual factors to optimize adherence and ensure that DHTs achieve their full potential in real-world settings.

In summary, future research should aim to establish standardized definitions and metrics for adherence to DHTs to improve consistency and comparability. Furthermore, while this review identified key factors influencing adherence, there is still a need for more research on the interaction between personal, technological, social, and contextual factors. Exploring these relationships in greater depth can inform the development of more comprehensive, integrative predictive models, translating broad findings into targeted strategies for developers, health care professionals, and policymakers to enhance adherence to DHT.

### Limitations

While this review offers valuable insights and is, to our knowledge, the first to systematically examine all factors influencing adherence across diverse populations and types of DHTs, several limitations must be acknowledged. Despite a broad search across 4 databases, the heterogeneity of study designs, populations, and DHTs may limit the generalizability of findings. The exclusion of non-English, Portuguese, and Spanish studies, as well as the restricted time frame, may have led to the omission of relevant studies and introduced potential language bias. Furthermore, restricting the review to peer-reviewed studies and the 3 selected languages may have excluded relevant gray literature and studies from non-English-speaking contexts, which are particularly important in the rapidly evolving field of digital health. This restriction also increases the risk of publication bias, as studies with negative or inconclusive findings are less likely to be published. Although the methodological quality of included studies was appraised using the JBI checklist, no formal assessment of publication or reporting bias was performed. This limits the ability to fully evaluate the presence of selective outcome reporting, thereby affecting the overall transparency and robustness of the findings. In addition, while our focus on statistically significant factors was intended to ensure clinical relevance and methodological rigor, this approach may have contributed to an overrepresentation of positive findings. By excluding nonsignificant findings, which may still hold exploratory value, the synthesis may underrepresent the full spectrum of evidence and inadvertently amplify the perceived strength of certain associations. Additionally, the lack of consistent definitions and standardized measures of adherence across studies complicates comparisons and limits the ability to draw firm conclusions.

Moreover, most included studies were conducted in Europe (n=25; [43-45,49-51,53,57,58,62,65-67,69,71,74-77,79,81,83,85,88,90]) and focused on mHealth apps (n=31; [8,40,44-53,57-60,65,68-71,74,76,77,79,81,87-91]). This may be explained by regional policy initiatives such as the Germany Digital Healthcare Act**,** which legally enables physicians to prescribe certified digital health applications reimbursed by statutory insurance [110]. Similarly, Belgium’s mhealthBelgium validation pyramid establishes a structured framework for assessing and reimbursing mHealth apps, including several that have achieved top-level funding status [111]*.*

### Conclusions

This systematic review offers a comprehensive overview of the factors influencing adherence to DHTs. The findings reveal that adherence is shaped by a complex interplay of determinants, which can be grouped into 4 key categories, such as personal factors, technology and intervention content factors, social and support system factors, and contextual factors. Among these, several stand out as particularly actionable, including digital literacy, perceived usefulness, accessibility, user experience, and social support from caregivers and health care professionals. These factors offer concrete targets for stakeholders. Developers can focus on usability and personalization of DHT, health care providers on guidance and encouragement, and policymakers on improving accessibility and digital literacy. Prioritizing these key determinants can help translate broad insights into targeted strategies to enhance sustained DHT adherence in real-world settings.

Among the theories and models identified, the UTAUT emerged as the most frequently applied. However, these were developed outside the digital health context and lacked specificity to capture key factors influencing long-term adherence to DHTs. The findings underscore the need for more integrative predictive models and frameworks, along with consistent definitions and measurement strategies of adherence, to guide future research and optimize the design and implementation of DHTs.

## Supplementary material

10.2196/77362 Multimedia Appendix 1 Search string. Filters applied: last 5 years, English, Portuguese, or Spanish and “humans.”

10.2196/77362 Multimedia Appendix 2 Quality assessment of the included studies using the Joanna Briggs Institute critical appraisal checklists.

10.2196/77362 Multimedia Appendix 3 Main findings and characteristics of the selected studies.

10.2196/77362 Checklist 1 PRISMA checklist.

## Abbreviations

DHT: digital health technology
DOI: diffusion of innovations theory
HBM: health belief model
JBI: Joanna Briggs Institute
MeSH: Medical Subject Headings
mHealth: mobile health
PICO: population, intervention, comparison, outcome
PMT: protection motivation theory
PRISMA: Preferred Reporting Items for Systematic Reviews and Meta-Analyses
RQ: research question
SCT: social cognitive theory
TAM: technology acceptance model
TFA: theoretical framework of acceptability
TIB: theory of interpersonal behavior
TPB: theory of planned behavior
TR: technology readiness
TRA: theory of reasoned action
TRAM: technology readiness and acceptance model
UTAUT: unified theory of acceptance and use of technology
WHO: World Health Organization
