# Supplementary material for: Understanding Adherence to Digital Health Technologies: Systematic Review of Predictive Factors
Source: J Med Internet Res. 2025 Nov 17;27:e77362. doi: 10.2196/77362 (PMC12622860; doi:10.2196/77362)
Supplement: Multimedia Appendix 1 [file jmir-v27-e77362-s001.docx]

Table S1. Search string. Filters applied: last 5 years, English, Portuguese or Spanish and “humans”.

| **Database** | **Search terms** | **Results (number of articles, n)** |
| --- | --- | --- |
| PubMed | ("tool*"[Title/Abstract] OR "measure*"[Title/Abstract] OR "questionnaire*"[Title/Abstract] OR "survey*"[Title/Abstract] OR "instrument*"[Title/Abstract] OR "scale*"[Title/Abstract] OR "theoretical model*"[Title/Abstract] OR "framework*"[Title/Abstract] OR "conceptual model*"[Title/Abstract] OR "determinant*"[Title/Abstract] OR "predictor*"[Title/Abstract] OR "barrier*"[Title/Abstract] OR "facilitat*"[Title/Abstract] OR "obstacl*"[Title/Abstract] OR "challeng*"[Title/Abstract] OR "imped*"[Title/Abstract] OR "obstruct"[Title/Abstract] OR "hindrance"[Title/Abstract] OR "interfer*"[Title/Abstract] OR "promot*"[Title/Abstract] OR "motiv*"[Title/Abstract] OR "driver*"[Title/Abstract] OR "stimulant*"[Title/Abstract] OR "enabl*"[Title/Abstract]) **AND** ("adheren*"[Title/Abstract] OR "nonadheren*"[Title/Abstract] OR "complian*"[Title/Abstract] OR "persisten*"[Title/Abstract] OR "noncomplian*"[Title/Abstract] OR "nonpersisten*"[Title/Abstract] OR "non complian*"[Title/Abstract] OR "non adheren*"[Title/Abstract] OR "non persisten*"[Title/Abstract]) **AND** ("digital health"[Title/Abstract] OR "digital health technology"[Title/Abstract] OR "eHealth" "e-Health"[Title/Abstract] OR "mHealth"[Title/Abstract] OR "mobile health"[Title/Abstract] OR "telemedicine"[Title/Abstract] OR "telehealth"[Title/Abstract] OR "digital intervention"[Title/Abstract] OR "health app"[Title/Abstract] OR "health technology"[Title/Abstract] OR "mobile app*"[Title/Abstract]) | n=2801  17.12.24 |
| PsycINFO | Title: (“tool*” OR “measure*” OR “questionnaire*” OR “survey*” OR “instrument*” OR “scale*” OR “theoretical model*” OR “framework*” OR “conceptual model*” OR “determinant*” OR “predictor*” OR “barrier*” OR “facilitat*” OR “obstacl*” OR “challeng*” OR “imped*” OR “obstruct” OR “hindrance” OR “interfer*” OR “promot*” OR “motiv*” OR “driver*” OR “stimulant*” OR “enabl*”) **AND** Title: (“adheren*” OR “nonadheren*” OR “complian*” OR “persisten*” OR “noncomplian*” OR “nonpersisten*” OR “non complian*” OR “non adheren*” OR “non persisten*”) **AND** Title: (“digital health” OR "digital health technology" OR "eHealth" “e-Health” OR "mHealth" OR "mobile health" OR "telemedicine" OR "telehealth" OR "digital intervention" OR "health app" OR "health technology" OR "mobile app*")  +  Abstract: (“tool*” OR “measure*” OR “questionnaire*” OR “survey*” OR “instrument*” OR “scale*” OR “theoretical model*” OR “framework*” OR “conceptual model*” OR “determinant*” OR “predictor*” OR “barrier*” OR “facilitat*” OR “obstacl*” OR “challeng*” OR “imped*” OR “obstruct” OR “hindrance” OR “interfer*” OR “promot*” OR “motiv*” OR “driver*” OR “stimulant*” OR “enabl*”) **AND** Abstract: (“adheren*” OR “nonadheren*” OR “complian*” OR “persisten*” OR “noncomplian*” OR “nonpersisten*” OR “non complian*” OR “non adheren*” OR “non persisten*”) **AND** Abstract: (“digital health” OR "digital health technology" OR "eHealth" “e-Health” OR "mHealth" OR "mobile health" OR "telemedicine" OR "telehealth" OR "digital intervention" OR "health app" OR "health technology" OR "mobile app*") | n= 12 + 509  17.12.24 |
| Scopus | TITLE-ABS-KEY ( "tool*" OR "measure*" OR "questionnaire*" OR "survey*" OR "instrument*" OR "scale*" OR "theoretical model*" OR "framework*" OR "conceptual model*" OR "determinant*" OR "predictor*" OR "barrier*" OR "facilitat*" OR "obstacl*" OR "challeng*" OR "imped*" OR "obstruct" OR "hindrance" OR "interfer*" OR "promot*" OR "motiv*" OR "driver*" OR "stimulant*" OR "enabl*" ) **AND** TITLE-ABS-KEY ( "adheren*" OR "nonadheren*" OR "complian*" OR "persisten*" OR "noncomplian*" OR "nonpersisten*" OR "non complian*" OR "non adheren*" OR "non persisten*" ) **AND**  TITLE-ABS-KEY ( ( "digital health" OR "digital health technology" OR "eHealth" "e-Health" OR "mHealth" OR "mobile health" OR "telemedicine" OR "telehealth" OR "digital intervention" OR "health app" OR "health technology" OR "mobile app*" ) | n=1446   17.12.24 |
| IEEE Xplore | ("Publication Title":"tool" OR "tools" OR "measure*" OR "questionnaire" OR "questionnaires" OR "survey" OR "surveys" OR "instrument" OR "instruments" OR "scale" OR "scales" OR "theoretical model" OR "theoretical models" OR "framework" OR "frameworks" OR "conceptual model" OR "conceptual models" OR "determinant" OR "determinants" OR "predictor" OR "predictors" OR "barrier" OR "barriers" OR "facilitators" OR "obstacles" OR "challenges" OR "impediments" OR "obstructions" OR "hindrance" OR "interference" OR "promotion" OR "motivation" OR "drivers" OR "stimulants" OR "enablers") **AND** ("Publication Title":"adherence" OR "nonadherence" OR "compliance" OR "persistence" OR "noncompliance" OR "nonpersistence") **AND** ("Publication Title":"digital health" OR "digital health technology" OR "eHealth" OR "e-Health" OR "mHealth" OR "mobile health" OR "telemedicine" OR "telehealth" OR "digital intervention" OR "health app" OR "health apps" OR "health technology")  +  ("Abstract":"tool" OR "tools" OR "measure*" OR "questionnaire" OR "questionnaires" OR "survey" OR "surveys" OR "instrument" OR "instruments" OR "scale" OR "scales" OR "theoretical model" OR "theoretical models" OR "framework" OR "frameworks" OR "conceptual model" OR "conceptual models" OR "determinant" OR "determinants" OR "predictor" OR "predictors" OR "barrier" OR "barriers" OR "facilitators" OR "obstacles" OR "challenges" OR "impediments" OR "obstructions" OR "hindrance" OR "interference" OR "promotion" OR "motivation" OR "drivers" OR "stimulants" OR "enablers") **AND** ("Abstract":"adherence" OR "nonadherence" OR "compliance" OR "persistence" OR "noncompliance" OR "nonpersistence") **AND** ("Abstract":"digital health" OR "digital health technology" OR "eHealth" OR "e-Health" OR "mHealth" OR "mobile health" OR "telemedicine" OR "telehealth" OR "digital intervention" OR "health app" OR "health apps" OR "health technology") | n= 103+161  17.12.24 |
| Total | | n= 5032 |
| Total after removing duplicates | | n=4031 |
