# Supplementary material for: Understanding Adherence to Digital Health Technologies: Systematic Review of Predictive Factors
Source: J Med Internet Res. 2025 Nov 17;27:e77362. doi: 10.2196/77362 (PMC12622860; doi:10.2196/77362)
Supplement: Multimedia Appendix 2 [file jmir-v27-e77362-s002.docx]

Table S2**.** Quality assessment of the included articles using the JBI critical appraisal checklists.

| **Author, Year, Country, Reference** | **Study Design** | **Critical Appraisal Tool Used** | **Score** | **Quality (high/moderate/low)** |
| --- | --- | --- | --- | --- |
| Addotey-Delove, M. et al, 2023, Multiple developing countries | Scoping review (n=85) | Checklist for Systematic Reviews | 6/11 | Moderate |
| Apergi, LA. et al, 2021, USA | cross-sectional study | Checklist for Analytical Cross-Sectional Studies | 8/8 | High |
| Armbruster, C. et al, 2022, Multiple countries | Scoping review (n=10) | Checklist for Systematic Reviews | 8/11 | Moderate |
| Arnold, C. et al, 2020, Australia | Qualitative study (semi-structured interviews) | Checklist for Qualitative Research | 6/10 | Moderate |
| Arsenijevic, J. et al, 2020, Multiple countries | Systematic review and meta-analysis (n=29) | Checklist for Systematic Reviews | 7/11 | Moderate |
| Balakrishnan, AS. et al, 2021, USA | Cohort study | Checklist for Cohort Studies | 8/11 | Moderate |
| Bartlett, YK. et al, 2021, England | Mixed-methods cross-sectional study (quantitative and qualitative -interviews) | Checklist for Analytical Cross-Sectional Studies + Checklist for Qualitative Research | 6/8 + 7/10 | Moderate |
| Böhm, AK. et al, 2020, Denmark | Cohort study | Checklist for Cohort Studies | 7/11 | Moderate |
| Brusniak, K. et al, 2020, Germany | Cohort study | Checklist for Cohort Studies | 10/11 | High |
| Chong, CJ. et al, 2024, Malaysia | Cross-sectional study | Checklist for Analytical Cross-Sectional Studies | 6/8 | High |
| Chong, CJ. et al, 2024, Malaysia | Qualitative study (semi-structured interviews) | Checklist for Qualitative Research | 8/10 | High |
| Colls, J. et al, 2020, USA | RCT | RCT Checklist | 9/13 | Moderate |
| d'Agate, D. et al, 2024, France | Cohort study | Checklist for Cohort Studies | 6/11 | Moderate |
| Dahlhausen F. et al, 2021, Germany | Mixed-methods cross-sectional study (quantitative and qualitative -semi-structured interviews) | Checklist for Analytical Cross-Sectional Studies + Qualitative Research | 6/8 + 7/10 | Moderate |
| Delestre, F. et al, 2023, France | Qualitative study (semi-structured interviews) | Checklist for Qualitative Research | 7/10 | Moderate |
| Dieciuc, M. et al, 2024, USA | Qualitative study (focus groups) | Checklist for Qualitative Research | 7/10 | Moderate |
| Gawałko, M. et al, 2022, European Countries | Cohort study | Checklist for Cohort Studies | 9/11 | High |
| Ouimet, AG. et al, 2020, Canada | Cross sectional study | Checklist for Analytical Cross Sectional Studies | 6/8 | High |
| Haldane, V. et al, 2019, Singapore | Mixed-methods cross-sectional study (quantitative and qualitative - semi-structured interviews) | Checklist for Analytical Cross Sectional Studies + Checklist for Qualitative Research | 6/8 + 7/10 | Moderate |
| Harst, L. et al, 2019, Multiple Countries | Systematic review (n=24) | Checklist for Systematic Reviews | 9/11 | High |
| Hasnan, S. et al, 2022, Multiple Countries | Systematic Review (n=5) | Checklist for Systematic Reviews | 7/11 | Moderate |
| Jakob, R. et al, 2022, Multiple Countries | Systematic review (n=99) | Checklist for Systematic Reviews | 7/11 | Moderate |
| Johansen, S. et al, 2023, Denmark | Qualitative study (think-aloud testing) | RCT Checklist | 8/10 | High |
| Jukic, T. et al, 2020, Slovenia | Cohort study | Checklist for Cohort Studies | 9/11 | High |
| Lee, J. and Trudel, R., 2023, USA | Randomized Experimental Study (5 studies) | Checklist for Quasi-Experimental Studies | 8/9 | High |
| Lee, M. et al, 2022, Korea | Cohort study | Checklist for Cohort Studies | 10/11 | High |
| Liptáková, S. et al, 2022, Multiple Countries | Systematic Review (n=69) | Checklist for Systematic Reviews | 6/11 | Moderate |
| Mandal, S. et al, 2022, USA | Secondary analysis RCT | RCT Checklist | 8/13 | Moderate |
| Moshe, I. et al, 2022, Germany | RCT | RCT Checklist | 9/13 | Moderate |
| Nelson, L. et al, 2020, USA | RCT | RCT Checklist | 9/13 | Moderate |
| Nordberg, B. et al, 2024, Kenya | Cohort study | Checklist for Cohort Studies | 10/11 | High |
| Øksnebjerg, L. et al, 2020, Denmark | Cohort study | Checklist for Cohort Studies | 9/11 | High |
| Patrascu, R. et al, 2021, Romania | Cross-Sectional Study | Checklist for Analytical Cross-Sectional Studies | 6/8 | High |
| Patrascu, R. et al, 2022, Romania | Cross-Sectional Study | Checklist for Analytical Cross-Sectional Studies | 6/8 | High |
| Renfrew, ME. et al, 2021, Australia and New Zealand | Qualitative study (questionnaires) | Checklist for Qualitative Research | 6/10 | Moderate |
| Rennie, K. et al, 2023, Fenland | Cohort study, mixed-methods (quantitative and qualitative – semi-structured interviews) | Checklist for Cohort Studies + Checklist for Qualitative Research | 7/10 + 7/10 | Moderate |
| Richterman, A. et al, 2023, USA | Qualitative Study (Semi-structured interviews) | Checklist for Qualitative Research | 7/10 | Moderate |
| Hermosa, JLR. et al, 2020, Spain | Cohort study | Checklist for Cohort Studies | 8/11 | Moderate |
| Rotondi, AJ. et al, 2024, USA | RCT | RCT Checklist | 9/13 | Moderate |
| Ruetsch, C. et al, 2021, USA | Cross-sectional study | Checklist for Analytical Cross-Sectional Studies | 8/8 | High |
| Sanchez-Ortuno, M. et al, 2023, France | Case Series Study | Checklist for Case Series | 6/10 | Moderate |
| Sassone B, et al, 2024, Italy | Cohort study | Checklist for Cohort Studies | 11/11 | High |
| Schroeder, T. et al, 2024, Germany | Qualitative study (semi-structured interviews) | Checklist for Qualitative Research | 9/10 | High |
| Schroeder, T. et al, 2024, Germany | Qualitative study (semi-structured interviews) | Checklist for Qualitative Research | 7/10 | Moderate |
| Schuttner, L. et al, 2024, USA | Cohort study | Checklist for Cohort Studies | 10/11 | High |
| Seppen, B. et al, 2023, Finland | Qualitative study (focus groups) | JBI Qualitative Tool | 7/10 | Moderate |
| Sharma, P. et al, 2024, USA | Cross-sectional study | Checklist for Analytical Cross-Sectional Studies | 8/8 | High |
| Siebenhüner, AR. et al, 2021, Switzerland | Cohort study | Checklist for Cohort Studies | 10/11 | High |
| Silva, CV. et al, 2022, Australia | RCT | RCT Checklist | 9/13 | Moderate |
| Slevin, P. et al, 2019, Dublin | Qualitative study (semi-structured interviews) | Checklist for Qualitative Research | 6/10 | Moderate |
| Sotirova, MB. et al, 2020, Multiple countries | Systematic review (n=11) | Checklist for Systematic Reviews | 8/11 | Moderate |
| Stone, C. et al, 2024, England | Qualitative study (questionnaire) | Checklist for Qualitative Research | 7/10 | Moderate |
| Thomas, BE. et al, 2020, India | Qualitative study (interviews) | Checklist for Qualitative Research | 7/10 | Moderate |
| Thomas, BE. et al, 2021, India | Qualitative study (interviews) | Checklist for Qualitative Research | 7/10 | Moderate |
| Thornton, L. et al, 2022, Australia | Cross-sectional analysis of RCT | Checklist for Analytical Cross-Sectional Studies | 8/8 | High |
| Touzani, R. et al, 2021, France | Cross-sectional study | Checklist for Analytical Cross-Sectional Studies | 8/8 | High |
| Vaghefi, I. et al, 2019, USA | Qualitative study (pre and post-use interviews) | Checklist for Qualitative Research | 7/10 | Moderate |
| Wyl, V. et al, 2021, Switzerland | Cross-sectional study | Checklist for Analytical Cross-Sectional Studies | 8/8 | High |
| Yang, X. et al, 2019, Multiple Countries | Scoping Review (n=24) | Checklist for Systematic Reviews | 7/11 | Moderate |
| Yang, Y. et al, 2022, Multiple Countries | Scoping Review (n=54) | Checklist for Systematic Reviews | 6/11 | Moderate |
| Zhang, Y. et al, 2020, China | Cross-sectional study | Checklist for Analytical Cross-Sectional Studies | 8/8 | High |
