# Supplementary material for: Understanding Adherence to Digital Health Technologies: Systematic Review of Predictive Factors
Source: J Med Internet Res. 2025 Nov 17;27:e77362. doi: 10.2196/77362 (PMC12622860; doi:10.2196/77362)
Supplement: Multimedia Appendix 3 [file jmir-v27-e77362-s003.docx]

Table S3**.** Main findings and characteristics of the selected articles

| **Author(s), year, country** | **Study design** | **Adherence concept** | **Theories, models or frameworks** | **Population characteristics** | **Type of DHT** | **Purpose of DHT** | **Factors influencing adherence concept** | **Adherence concept definition/measurement** |
| --- | --- | --- | --- | --- | --- | --- | --- | --- |
|  |  |  |  |  |  |  |  |  |
| Addotey-Delove, M. et al, 2023, Multiple developing countries [26] | Scoping review (n=85) | Adoption | NR | healthcare workers | mHealth | NR | Engagement and funding  Training and technical support  Infrastructure  System utility  Motivation and staffing  Cost and ownership of a mobile phone | NR |
| Apergi, LA. et al, 2021, USA [51] | Cross-sectional study | Engagement | NR | HF patients (≥18 years); (n=47) | - Conversational agent | Uses a voice interface to ask questions related to HF treatment and symptoms and provide feedback | Higher age (↑)  Higher number of medications to manage HF (↓)  Black patients compared to non-Black patients (↓) | Number of days during the study period (90 days) in which the patient used the voice interface to answer the questionnaire. |
| Armbruster, C. et al, 2022, Multiple countries [45] | Scoping review (n=10) | Adherence | NR | Cancer patients | - Apps | For symptom reporting, mental health, quality of life, behaviour monitoring, health promotion, illness education, social support, community building, and personalized medication scheduling | Sociodemographic variables (age, gender, education level, relationship status, employment status)  Cancer-related factors and others (presence of comorbidities) | Varied across included studies. |
| Arnold, C. et al, 2020, Australia [46] | Qualitative study (semi-structured interviews) | EngagemenE Engagement | NR | Adults with psychosis (18-65 years); (n= 17) | - Web-based program | promote self-management of mental health and personal recovery | User-related factors (eg, mental health, personal circumstances, approach to using the website)  Users’ experience of the intervention (eg, having experienced similar content previously or finding the material confronting). | NR |
| Arsenijevic, J. et al, 2020, Multiple countries [6] | Systematic review and meta-analysis (n=29) | Adherence | NR | Vulnerable groups (older adults, chronically sick, minorities, people with low socioeconomic status, and migrants) | - eHealth tools | For several healthcare areas | Facilitators:  eHealth tools with multimodal content and direct patient-provider interaction | Defined as the number of people who are repetitive users (ie, use the eHealth tool more than once). |
| Balakrishnan, AS. et al, 2021, USA [89] | Cohort study | Engagement | NR | Patients undergoing prostate biopsy; (n=1025) | - text message-based | Provides health reminders and education programs for patients undergoing prostate biopsy | Single or divorced compared to married and/or partnered patients (↓)  Patients for whom English was not their primary language (↓) | Defined as a patient clicking a link delivered via SMS to access educational modules. |
| Bartlett, YK. et al 2021, England [72] | Mixed-methods cross-sectional study (quantitative and qualitative -interviews) | Acceptance | TFA | Adults (≥18 years) with self-reported type 2 diabetes; (n=107) | - text message-based | Promote medication adherence for people with type 2 diabetes | Affective attitude  Burden  Ethicality  Intervention coherence  Opportunity costs  Perceived effectiveness  Self-efficacy | Calculated based on participants' ratings. Each message was rated on three aspects: cognitive (easy vs. hard), emotional (like vs. dislike), and appropriateness (useful vs. not useful). |
| Böhm, AK. et al, 2020, Denmark [52] | Cohort study | Engagement | NR | Diabetes patients; (n=9051) | - App | Patient support app for diabetes | Women, except in the exercise module (↓)  Older age, except the glucose monitoring module (↑) | Defined as progress comprising 4 distinct stages: point of engagement, period of sustained engagement, disengagement, and reengagement. |
| Brusniak, K. et al, 2020, German [65] | Cohort study | Compliance | NR | Pregnant women; (n= 585) | - App | Self-tracking during pregnancy | Higher income (↑)  Higher education (↑)  Public health insurance (↑)  Origin (other versus German) (↑)  Smoking (↓) | Defined as an individual completion rate ≥80%. |
| Chong, CJ. et al, 2024, Malaysia [75] | Cross-sectional study | Adoption | TRAM | Patients with diabetes type 2 (≥18 years); (n= 400) | - App | To promote medication adherence management for diabetes type 2 | low awareness (↓)  lack of need (↓)  poor digital literacy (↓)  low learning motivation (↓)  vision problem (↓)  cost of app subscription (↓)  device availability and limited storage (↓) | NR |
| Chong, CJ. et al, 2024, Malaysia [76] | Qualitative study (semi-structured interviews) | Adoption | TRAM | Patients with diabetes type 2 (≥18 years); (n= 25) | - App | To promote medication adherence management for diabetes type 2 | Barriers adoption:  -patient elements (low awareness, lack of need, poor digital literacy, low learning motivation, vision problem);  -smartphone elements (no availability of device, limited storage);  -Monetary element (higher cost) | NR |
| Colls, J. et al, 2020, USA [53] | RCT | Adherence | NR | Patients with rheumatoid arthritis (RA) (≥18 years ); (n=78) | - App | To allow patients to communicate with their clinicians and track the activity of RA | Age ≥ 65 (↑)  Low baseline Clinical  Disease Activity Index (better disease control) (↑) | Defined by assessing the response rate for all ePRO questionnaires, as well as for specific ePRO domains. The rate was estimated for the entire study period. |
| d'Agate, D. et al, 2024, France [110] | Cohort study | Compliance | NR | Patients undergoing uro-oncological surgery (n=24) | - App | Allows the all-in-one implementation of an optimised pathway, gathering prehabilitation and rehabilitation protocols, patient education, checklists, ePRO collection, and remote monitoring. | Older age (64 vs. 61 years) (↓) | Defined by responses to their digital questionnaires and/or reading the digital materials at the expected frequency. |
| Dahlhausen F. et al, 2021, German [95] | Mixed-methods cross-sectional study (quantitative and qualitative -semi-structured interviews) | Adoption | NR | General practitioners, physicians, and psychotherapists; (n=1308) | - Apps | Support the detection, monitoring,  treatment, mitigation, or compensation of disease, injury, or disability. | Facilitators:  - Additional information about apps  -recommendations by medical associations  -positive experience reports about apps from medical colleagues  -opportunities to test apps  - increased reimbursement for medical services related to the apps | NR |
| Delestre, F. et al, 2023, France [92] | Qualitative study (semi-structured interviews) | Adherence | NR | cancer survivors (≥55 years); (n=15) | - Web app | Offers nutrition and physical  activity coaching to cancer survivors | Facilitators:  - social relations;  -supportive family  -trusted prescriber  -continuity in the care pathway  -appropriate choice of design features | NR |
| Dieciuc, M. et al, 2024, USA [60] | Qualitative study (focus groups) | Engagement | NR | - older adults (≥60 years); (n=21) - and younger adults (18-25 years); (n=21) | - Apps | - Facilitating early detection and preventing or reversing cognitive decline | - Facilitators: - -ease of use (broadly construed as flexibility, convenience, and usability) - -reminders - Barriers: - -sheer forgetfulness. - -lack of social engagement - -lack of seeing tangible improvments | - NR |
| Gawałko, M. et al, 2022, European Countries [54] | Cohort study | Adherence | NR | Patients with diagnosed atrial fibrillation (AF); (n=990) | - App | To provide ongoing management and comprehensive care to patients with AF during the COVID-19 pandemic lockdown within cardiology centres in Europe. | Older age and absence of diabetes (↑)  older age (↑)  female sex (↑)  previous AF ablation (↑) | Defined as the number of measurements per number of expected measurements (at least 3 daily) over the entire prescription period. |
| Ouimet, AG. et al, 2020, Canada [82] | Cross-sectional study | Intention to continue use | NR | Users; (n= 178) | - teleconsultation plataform | Offers remote healthcare to employees and their families through chat or video calls. AI performs an initial assessment, and if needed, a nurse provides further consultation, leading to a diagnosis, a follow-up with a Dialogue physician, or a referral to another healthcare provider | Confirmation of expectations (↑)  Usefulness (↑)  Quality (↑) | NR |
| Haldane, V. et al, 2019, Singapore [47] | Mixed-methods cross-sectional study (quantitative and qualitative - semi-structured interviews) | Adherence | UTAUT | Adults with ASCVD or risk factors (≥40 years); (n=20 for the interviews and n=100 surveys) | text message-based | to support patient adherence to medications for ASCVD. | Therapy-related factors (complexity of medication regime, duration of therapy, inconvenience with lifestyle, adverse effects)  Condition-related factors | NR |
| Harst, L. et al, 2019, Multiple Countries [83] | Systematic review (n=24) | Acceptance | DFM  HBM  PMT  TR  SCT  TAM2  TIB  TPB  TRA  DOI  UTAUT  TAM | Patients, social environment (relatives and peers or peer groups),  and health care providers | - telemedicine | NR | Perceived usefulness  Social influences  Attitude | NR |
| Hasnan, S. et al, 2022, et al, Multiple Countries [30] | Systematic Review (n=5) | Adherence | NR | Older patients (≥65 years old) with cancer | - digital health interventions | NR | Usability  Perceived usefulness | NR |
| Jakob, R. et al, 2022, Multiple Countries [8] | Systematic review (n=99) | Adherence | NR | Adults (≥18 years old) | - Apps | To prevent or manage NCDs | -Intervention-related factors (e.g. personalization of the content, reminders in the form of individualised push notifications, user-friendly and technically stable app design, and personal support, social and gamification features).  -Patient-related factors (e.g. user characteristics or recruitment channels ) | Calculated as the ratio between the estimated intended use and actual use. |
| Johansen, S. et al, 2023, Denmark [91] | Qualitative study (think-aloud testing) | Adherence | NR | Adolescents with knee pain; (n=12) | - App | To promote self-management of knee pain | -user experience and feedback (installation, goal setting, feedback on information videos, feedback on self-tracking, and feedback on treatment videos)  -contextual challenges (parental inclusion and hiding the knee pain) | NR |
| Jukic, T. et al, 2020, Slovenia [111] | Cohort study | Adherence | NR | Female healthcare workers; (n=80) | - Web App | To improve and modify lifestyle to diminish stress | No factors or participant’s characteristics were identified to affect the adherence | Defined based on the number of log-ins into the web-based application. |
| Lee, J. and Trudel, R., 2023, USA [61] | Randomized Experimental Study (5 studies) | Adoption | NR | Study 1: 304 participants;  Study 2: 209 male participants;  Study 3A: 396 male participants;  Study 3B: 403 male participants;  Study 3C: 607 male participants | - Apps | To promote mental health | Men (↓)  Perceived “masculine men” (mental health-feminine stereotype) (↓) | NR |
| Lee, M. et al, 2022, Korea [84] | Cohort study | Adoption and compliance | NR | Cancer patients (n=580) | - App | Electronic patient-reported outcome measure (ePROM). For patients to report symptoms during cancer treatment | Greater expectations regarding the ease of use and usefulness (↑) (Adoption)  Greater satisfaction with usefulness (↑) (Compliance) | Adoption was considered good if the patient used the app voluntarily again after installation at a scheduled period. A patient was considered as having “good com  pliance” if continued to use the app following the instructions (reporting symptoms regularly and keeping a 7-day interval (± 2 days) for 21 days). |
| Liptáková, S. et al, 2022, Multiple Countries [31] | Systematic Review (n=69) | Adherence | NR | NR | - eHealth | mindfulness based programs (MBPs) to positively impact mental health | Demographic/personal predictors and psychological predictors | Varied across included studies. |
| Mandal, S. et al, 2022, USA [55] | Secondary analysis RCT | Engagement | NR | Type 2 diabetes patients; (n=61) | - mHealth | Patient-reported outcomes (PRO). For type 2 diabetes management | Older, hispanic descent, bilingual, and had a graduate degree (↑)  African American patients who reported the lowest annual income (↓) | Defined by response rate (number of messages that received a valid response × 100 number of messages sent by the program with questions on PRO). Other engagement measures were also used and analyse. |
| Moshe, I. et al, 2022, German [59] | RCT | Dropout | NR | Patients with chronic back pain (age≥18); (n= 253) | - digital health intervention | treatment and prevention of depression in individuals with chronic back pain | Lower level of education (↑)  Age (↔) | Defined as completing <6 modules. |
| Nelson, L. et al, 2020, USA [69] | RCT | Engagement | NR | Type 2 diabetes patients; (n=248) | - text message-based | supporting diabetes self-care | Black race (↓)  Worse baseline medication adherence and HbA1c (↓) | Defined as any response to the interactive text messages. |
| Nordberg, B. et al, 2024, Kenya [67] | Cohort study | Adherence | NR | Pregnant women living with HIV (age ≥ 18 years); n=299 | - text message-based | prevention of mother-to-child transmission of HIV | Having secondary education compared to primary education or less (↓)  Younger age (18–24 years) compared to being 35–44 years (↑)  Women who had disclosed  their HIV status (↑) | Defined as the rate of responding to text messages. |
| Øksnebjerg, L. et al, 2020, Denmark [94] | Cohort study | Adoption | NR | People living with dementia (n=112) and caregivers (n=98) | - App | To support memory and structure in daily living. Persons with dementia had access to a personal user account, and family caregivers were given a parallel login. | Shorter time from diagnosis (↑)  Caregiver activating the app (↑) | Defined as a minimum period of 90  days between the first and last use of the app. |
| Patrascu, R. et al, 2021, Romania [71] | Cross-sectional Study | desirability, acceptability and adherence | QTelemeDiab | Diabetes patients (n=114) | - Telemedicine | To improve diabetes care | Particular preferences regarding  telemedicine use, socio-economic, demographic,  disease history, occupational characteristics, management of diabetes | Measured as a score of the QtelemeDiab. |
| Patrascu, R. et al, 2022, Romania [96] | Cross-sectional Study | desirability, acceptability and adherence | QTelemeDiab | Diabetes patients (n=114) | - Telemedicine | To improve diabetes care | The presence of severe depression (↓)  The presence of severe anxiety (↓) | Measured as a score of the QtelemeDiab. |
| Renfrew, ME. et al, 2021, Australia and New Zealand [48] | Qualitative study (questionnaires) | Adherence | NR | Adults (age ≥ 18 years); (n=320) | - Web- and app-based | Mental health promotion intervention. To attenuate the risk of mental health distress | Facilitators:  -engaging content  -time availability and management,  -ease of accessibility  -easy or enjoyable practical challenges  -high perceived value  -personal motivation to complete the intervention.  Barriers:  -lack of time.  -completing and recording practical activities  - length of video content  -technical difficulties  -combination of personal factors | NR |
| Rennie, K. et al, 2023, Fenland [56] | Mixed-methods cohort study (quantitative and qualitative – semi-structured interviews) | Engagement | NR | Adults (44-70 years); (n=2524) | - App | tracking COVID-19 digital biomarkers (oxygen saturation, body  temperature, and resting heart rate) and symptoms | Older age groups (↑)  Lower managerial (↑)  Intermediate occupations (↑)  Working (↓)  Being a current smoker (↓)  being overweight or obese (↓)  Facilitators:  - Routine  - personal motivation  Barriers:  -high perceived stress  -user error  -app or equipment malfunctions preventing data input | Categorised by median weekly frequency of completing the 3 digital biomarker modules (for quantitative study). |
| Richterman, A. et al, 2023, USA [87] | Qualitative Study (Semi-structured interviews) | Acceptance | NR | Women opioid use disorder (OUD); (n=20) | - App | improves retention and abstinence for people with OUD. | Facilitators:  -provide new knowledge  -user-friendliness and app functions smoothly  Barriers:  -phone and internet access. | NR |
| Hermosa, JLR. et al, 2020, Spain [73] | Cohort study | Compliance | NR | Patients with COPD; (n=116) | - App | Recording daily symptoms as a useful strategy to detect COPD exacerbations | active smokers (↓)  greater dyspnea (↓)  diagnosis of depression (↓)  obesity (↓) | Defined as the percentage of available days that data were recorded in the app. |
| Rotondi, AJ. et al, 2024, USA [66] | RCT | Discontinue the use | NR | People with depressive or anxiety symptoms; (n=603) | - Web-based program | e-mental health treatment programs: evidence-based computerised cognitive behavioral therapy (CCBT). | less formal education (↑)  lower severity of anxiety symptoms (↑) | Participants were stratified by whether they “continued” the use of CCBT (yes or no), as those who completed at least one CCBT module. Those who did not complete the first CCBT module were categorised as “discontinued” use. |
| Ruetsch, C. et al, 2021, USA [62] | Cross-sectional study | Adoption | NR | psychiatric prescribers; (n=131) | - ingestible event marker (IEM) platform | Ingestible microsensors to help prescribers discern between medication non-adherence and medication ineffectiveness when considering treatment  tion for patients whose symptoms are not well controlled | Female gender (↑)  Perspectives on the value of adherence (↑) | Measured through the survey. |
| Sanchez-Ortuno, M. et al, 2023, France [112] | Case Series Study | Adherence | NR | Adults (≥ 18 years); (n=824)  . | - App | To manage insomnia complaints | Larger pre-intervention improvements (↑)  Higher levels of trust in the virtual agent (↑)  Better acceptance of the app (↑) | Evaluated via self-report. Users were asked with yes/no questions if they had followed each one of these recommendations. The total number of “yes” answers was taken as a quantitative indicator of adherence. Values ranged from 0 to 3. |
| Sassone B, et al . 2024, Italy [64] | Cohort study | Adherence | NR | Patients with ischemia; (n=252) | - Telemedicine | Web-based health  educational meeting (WHEM) after hospital discharge, as part of a secondary prevention program | Presence of a caregiver with digital proficiency (↑)  Higher education level (↑)  Increasing age (↓)  Female sex (↓) | NR |
| Schroeder, T. et al, 2024, German [81] | Qualitative study (semi-structured interviews) | Adoption | UTAUT  HBM | Physicians (n=28) and potential patients ((n=30); (≥ 50 years)) | - Apps | NR | Barriers:  - lack of information regarding mHealth apps and their positive health impacts  - physicians’ self-perceived digital competence and their evaluation  of patients’ digital proficiency | NR |
| Schroeder, T. et al, 2024, German [77] | Qualitative study (semi-structured interviews) | Adoption | NR | Doctors (n=28) | - Apps | NR | Operation and stability of the app  User experience  Benefits of the app  Cost of the app  Advocacy  Personal attitudes towards technology  Existing workload | NR |
| Schuttner, L. et al, 2024, USA [70] | Cohort study | Engagement | NR | Patients at high risk of hospitalization and mortality (n=1383070) | - telehealth modalities (telephone, video visits, and secure messaging) | NR | Black non-Hispanic individuals with greater comorbidity burdens (sustained or only transiently engaged) (↑) | Defined across 3-year period: never users (no telehealth use in any year); transient users (telehealth use in pandemic year 1 only); new persistent users (telehealth use in pandemic years 1 and 2); consistent users (telehealth use in all 3 years); and remaining as all others. |
| Seppen, B. et al, 2023, Finland [85] | Qualitative study (focus groups) | Adherence | NR | Patients with inflammatory arthritis; (n=22) | - App | to telemonitoring disease activity with electronic patient-reported outcomes (ePROs) | Facilitators:  -maximize perceived benefits (ability to skip (unneeded) physical consultations and discuss with clinicians the completed ePRO)  -reduce the effort to report ePROs (tailoring the frequency based on the patient) | NR |
| Sharma, P. et al, 2024, USA [57] | Cross-sectional study | Persistence | NR | Adults (≥18 years); (n=321) | - Video appointments services | NR | Older (↑)  Lived in rural areas (↑)  Not having a disability, handicap, or chronic disease (↑)  Personal beliefs (e.g. discomfort with video communication, difficulty interpreting nonverbal cues) (↓) | Measured as the response to the question “Have you ever had a video appointment with a healthcare provider?. The respondents who marked “no” were defined as “persistent nonusers". |
| Siebenhüner, AR. et al, 2021, Switzerland [74] | Cohort study | Adherence | NR | Cancer patients (≥18 years); (n=83) | - App | To deliver mindfulness and relaxation interventions for cancer patient | Decrease in distress (↓)  Moderate distress or an increase  in distress (↑) | Defined as the use of the app exercises regularly (at least once per week). |
| Silva, CV. et al, 2022, Australia [93] | RCT | Engagement | NR | Adults (18-40 years); (n=375) | - Text-message based | To promote sun protection behaviours | Personalised, but not interactive messaging (↓)  Interactive messages (↑)  Increasing frequency (compared to constant or decreasing frequency) (↓) | Defined as the number of messages responded to by a participant divided by the number of messages received during an intervention period. |
| Slevin, P. et al 2019, Dublin [78] | Qualitative study (semi-structured interviews) | Adoption | NR | COPD patients; (n=30) | - DHTs | NR | Barriers:  -lack of perceived usefulness  -digital literacy  - illness perception  -social context  Facilitators:  -existing digital self-efficacy  -personalised education  -community-based support | NR |
| Sotirova, MB. et al, 2020, Multiple countries [86] | Systematic review (n=11) | Acceptance | NR | Adults (≥18 years); at least 2/3 having received surgical intervention  for any type of cancer | - web-based program | exercise-based online self-management programmes for post-surgical cancer rehabilitation | Facilitators:  Interventions which were seen as time and cost-efficient, requiring acquisition of minimal or no new skills, which used coherent language, or which provided tailored information | Varied across included studies. |
| Stone, C. et al, 2024, England [90] | Qualitative study (questionnaires) | Acceptance | NR | Adults (18 -70 years); daily cigarette  smokers actively seeking to quit | - Wearable | smartwatch-based  relapse prevention system that uses passive detection of smoking to trigger just-in-time smoking cessation support | Barriers:  Bulkiness and size of the device  Notification style  Limitations of battery capacity | Assessed using a Qualtrics questionnaire with closed-ended  and free-text responses. |
| Thomas, BE. et al, 2020, India [79] | Qualitative study (interviews) | Acceptance | UTAUT | TB patients (n=62) and health  care providers (HCP) (n=31) | - cell phone-based | For monitoring tuberculosis medication adherence | Facilitators for patients:  -improved patient-HCP relationships  -TB pill-taking habit formation  -reduced need to visit health facilities (performance expectancy);  -improved family involvement in TB care (social influences);  - HCPs to engage positively in patients’ care (facilitating conditions)  Barriers for patients:  -reduced face-to-face contact with HCPs (performance expectancy);  -problems with cell phone access, literacy, cellular signal, or technology fatigue (effort expectancy)  -high TB- and HIV-related stigma within the family (social influences)  -poor counselling in the solution by HCPs (facilitating conditions).  Facilitators for HCP:  -perceptions that the solution improves the quality and efficiency of care and the patient-HCP relationship (performance expectancy)  - that the dashboard is easy to use (effort expectancy);  -leads to better coordination among HCPs (social influences).  Barriers for HCP:  -inadequate training of HCPs in the solution  -unequal changes in workload  -shortages of material (envelopes) | NR |
| Thomas, BE. et al, 2021, India [88] | Qualitative study (interviews) | Acceptance | UTAUT | Patients with multidrug-resistant tuberculosis (n=65) and  HCP (n=10) | - digital pillbox | Digital pillbox that provides pill-taking reminders and facilitates the remote monitoring of medication adherence | Facilitators for patients:  - audible and visual reminders  -remote monitoring reduced  the frequency of clinic visits (performance expectancy)  -device’s organization and labelling of medications made it easier  to take them correctly (effort expectancy)  -the device facilitated positive family involvement in the patient’s care (social  influences)  -remote monitoring made patients feel more cared for by the health system (facilitating conditions).  Barriers for patients:  -problems with the durability of the device  -difficulties with portability and storage (effort expectancy)  -concerns regarding stigma and the disclosure of patients’ diagnoses (social influences)  -incorrect understanding of the device because of suboptimal counselling  (facilitating conditions).  Facilitators for HCP:  -fewer in-person interactions  with patients, allowed HCPs to dedicate more time to other tasks, which improved job satisfaction. | NR |
| Thornton, L. et al, 2022, Australia [63] | Cross-sectional analysis of RCT | Engagment | NR | Students (11-14 years); (n=3610) | - App | Self-monitoring app for adolescents targeting six key health risk behaviours | Teacher prompts (↑)  Living in a major city (↑)  Female (↑) | Measured by app access. |
| Touzani, R. et al, 2021, France [58] | Cross-sectional study | Acceptance | NR | Adults (18-75 years); (n=1003) | - App | Monitoring contacts of patients with COVID-19 | Lower financial deprivation (↑)  Higher perceived usefulness (↑)  Age over 60 years (↑)  Trust in political representatives (↑)  Feeling concerned about the pandemic situation (↑)  Knowledge about the transmission of COVID-19 (↑) | Measured by a single question, the responses to which were grouped into three modalities: app-supporting, app-willing, and app-reluctant. |
| Vaghefi, I. et al, 2019, USA [80] | Qualitative study (pre and post-use interviews) | Continue use | NR | University members; (n=17) | - Apps | NR | Users’ assessment of mHealth app and its capabilities (user experience)  User’s persistence at their health goals (intent) | Acessed by the response to the question “please describe your  interactions with the app today” (eg, How many times and how  long you used it? What features did you utilize?). |
| Wyl, V. et al, 2021, Switzerland [68] | Cross-sectional study | Acceptance | NR | Participants (n=1511) | - App | Digital proximity tracing app to mitigate the transmission of SARS-CoV-2 | Higher monthly household income (↑)  More frequent internet use (↑)  Better adherence to recommendations for wearing masks (↑)  Nonsmoker status (↑)  Citizenship status (non-Swiss citizenship vs. Swiss) (↓)  Language region (French vs Swiss German) (↓)  Higher levels of trust in government and health authorities (↑) (subsample n=712) | Accessed as individuals who reported they used the app permanently or who turned it off  only occasionally - “app users”. Individuals who reported not using the app (either with or without an intention to do so later) were  considered “app nonusers”. |
| Yang, X. et al, 2019, Multiple Countries [49] | Scoping Review (n=24) | Adherence | NR | NR | - App | Promote and deliver physical activity | Personal Factors  Technology Features  Contextual Factors | Defined as four dimensions: frequency of app usage, intention/motivation to  sustain use of the app, degree of function use within the app, and the duration of app usage. |
| Yang, Y. et al, 2022, Multiple Countries [50] | Scoping Review (n=54) | Adherence | NR | Adults (≥18 years) | - mHealth | Promote and deliver physical activity | Users’ characteristics  Technology-related factors  Contextual factors | Varied across included studies. |
| Zhang, Y. et al, 2020, China [113] | Cross-sectional study | Adherence | NR | Hypertension participants (≥60 years); (n=212) | - Wearable | Blood pressure (BP) monitor device. Promotion of hypertension management | lower lifestyle compliance (↑)  lower medication compliance (↑)  higher total hypertension compliance (↑) | Defined as daily recording of BP data during the 30-day assessment period. |

NR - Not reported; (↑) - Statistically significant positive effect; (↓) - Statistically significant negative effect; (↔) - Significant but inconsistent effect; ePRO- electronic patient-reported outcomes; TFA - Theoretical Framework of Acceptability; TRAM - Technology Readiness and Acceptance Model; DFM - Dual Factor Model; HBM - Health Belief Model; PMT-Protection Motivation Theory; TR - Technology Readiness; SCT - Social Cognitive Theory; TAM2 - Technology Acceptance Model 2; TIB - Theory of Interpersonal Behavior; TPB - Theory of Planned Behavior; TRA - Theory of Reasoned Action; DOI - Diffusion of Innovations Theory; UTAUT - Unified Theory of Acceptance and Use of Technology; TAM - Technology Acceptance Model; QTelemeDiab -instrument for assessing patients’ desirability, acceptability, and adherence to telemedicine in diabetes.
